# Commissioning results of an automated treatment planning verification system

**DOI:** 10.1120/jacmp.v15i5.4838

**Published:** 2014-09-08

**Authors:** Christopher L. Nelson, Bryan E. Mason, Ronald C. Robinson, Kelly D. Kisling, Steven M. Kirsner

**Affiliations:** ^1^ Department of Radiation Physics The University of Texas MD Anderson Cancer Center, Physicians Network Houston TX USA

**Keywords:** dose verification, quality assurance, patient specific quality assurance

## Abstract

A dose calculation verification system (VS) was acquired and commissioned as a second check on the treatment planning system (TPS). This system reads DICOM CT datasets, RT plans, RT structures, and RT dose from the TPS and automatically, using its own collapsed cone superposition/convolution algorithm, computes dose on the same CT dataset. The system was commissioned by extracting basic beam parameters for simple field geometries and dose verification for complex treatments. Percent depth doses (PDD) and profiles were extracted for field sizes using jaw settings $3\;\times \;3\;{\text{cm}}^{2}\;‐\;40\;\times \;40\;{\text{cm}}^{2}$ and compared to measured data, as well as our TPS model. Smaller fields of $1\;\times \;1\;{\text{cm}}^{2}$ and $2\;\times \;2\;{\text{cm}}^{2}$ generated using the multileaf collimator (MLC) were analyzed in the same fashion as the open fields. In addition, 40 patient plans consisting of both IMRT and VMAT were computed and the following comparisons were made: 1) TPS to the VS, 2) VS to measured data, and 3) TPS to measured data where measured data is both ion chamber (IC) and film measurements. Our results indicated for all field sizes using jaw settings PDD errors for the VS on average were less than 0.87%, 1.38%, and 1.07% for 6x, 15x, and 18x, respectively, relative to measured data. PDD errors for MLC field sizes were less than 2.28%, 1.02%, and 2.23% for 6x, 15x, and 18x, respectively. The infield profile analysis yielded results less than 0.58% for 6x, 0.61% for 15x, and 0.77% for 18x for the VS relative to measured data. Analysis of the penumbra region yields results ranging from 66.5% points, meeting the DTA criteria to 100% of the points for smaller field sizes for all energies. Analysis of profile data for field sizes generated using the MLC saw agreement with infield DTA analysis ranging from 68.8%–100% points passing the 1.5%/1.5 mm criteria. Results from the dose verification for IMRT and VMAT beams indicated that, on average, the ratio of TPS to IC and VS to IC measurements was 100.5 ± 1.9% and 100.4 ± 1.3%, respectively, while our TPS to VS was 100.1 ± 1.0%. When comparing the TPS and VS to film measurements, the average percentage pixels passing a 3%/3 mm criteria based gamma analysis were 96.6 ± 4.2% and 97 ± 5.6%, respectively. When the VS was compared to the TPS, on average 98.1 ± 5.3% of pixels passed the gamma analysis. Based upon these preliminary results, the VS system should be able to calculate dose adequately as a verification tool of our TPS.

PACS number: 87.55.km

## I. INTRODUCTION

In radiation oncology, a secondary check of the treatment planning system's (TPS) dose calculations is common practice.^1^, ^2^, ^3^, ^4^ Secondary independent calculations can be performed by hand or using a commercial software package. These calculations are typically based upon reference data measured in a water phantom, and often rely on a primary component and a scatter component for the dose calculation.^(5)^ A large portion of patients are treated using either intensity‐modulated radiation therapy (IMRT) or volume‐modulated arc therapy (VMAT). During these treatments, many small irregularly shaped beam apertures are used during each treatment field, making a secondary calculation by hand impractical. Thus, the primary dose calculation algorithm is commonly verified for each patient using measurement‐based techniques.^6^, ^7^, ^8^, ^9^, ^10^, ^11^, ^12^ Alternatively, the dose calculation algorithm can be verified with software.^2^, ^13^ These systems typically compare point dose computed by the TPS. These calculation algorithms work well when good conditions exist; however, when this calculation point falls behind a closed MLC leaf, or is placed near a region having a large density heterogeneity, these algorithms may not accurately compute the dose to the point. Clinically, an explanation of this failure or movement of the calculation point in order to satisfy clinical QA criteria would be required.

A superior solution would have a single system that can perform an independent check on both simple and complex geometry cases, that accounts for heterogeneities, angle of incidence at beam entry point, and that would use a full 3D scatter algorithm to compute dose.^(14)^ While Monte Carlo methods could satisfy these criteria,^(15)^ computation time and hardware are often limiting factors. While this has been performed using a separate TPS to validate the original TPS, no such commercial TPS verification system (VS) has been validated. Thus, a commercial treatment planning and dose delivery verification system (Mobius 3D, v1.3.1, Mobius Medical Systems, Houston, TX) was acquired as a secondary verification tool of our TPS and satisfies all of these requirements. This utility reads in DICOM RT plans, CT datasets, and RT dose from the treatment planning system, performs a 3D dose calculation using a collapsed cone convolution superposition (CCCS) algorithm. The VS dose calculation algorithm is implemented using the graphical processing unit (GPU), which is capable of performing faster calculations than the central processing unit (CPU) of the computer. By doing so, the VS is able to make less assumptions than a traditional CPU‐based CC algorithm. Following dose computation, the VS compares the dose calculated in the TPS to its dose, and the system performs a 3D gamma calculation to compare the two dose distributions. Comparisons are done automatically as soon as it receives all the data from the DICOM RT exported from the TPS. Several other key features that are not tested in this study, but will be addressed in future works, including testing that the VS is configurable to check dose‐volume histogram (DVH) values against user defined levels to ensure dose to both the target structures and avoidance structures are within criteria. The VS is also capable of detecting possible machine/patient collisions in geometries that may place the patient close to the gantry head. The goal is to have a single VS that can perform a secondary check on our TPS for both simple and complex cases. The VS utilizes reference beam models for existing linear accelerators. If the measured beam data agrees with the manufacturers golden data, the stock reference model can be used. All that is needed is the absolute output at 10 cm depth. If the measured data varies from the standard manufacturers data, depth dose points, output factors, and off axis ratios for your machine can be entered and the VS will then scale the reference beam data. The beam model will then be updated for each machine through the automodeling function. Since the VS uses an independently developed collapsed cone superposition algorithm and linear accelerators may not match standard beam data, the accuracy of the dose calculation must be commissioned and validated.^(16)^ The purpose of this work was to present the methodology and results from the commissioning process of this system, when the nonstock beam model is used with the intent of using it as a TPS verification system.

## II. MATERIALS AND METHODS

The commissioning process for a TPS verification system was done in several steps, each of which tests the system in a more complex environment. To begin, percent depth dose (PDD) and profiles were extracted from the VS, the TPS (Pinnacle^3^, Philips Healthcare, Andover, MA), and measured data acquired at the time of beam modeling and commissioning of our linear accelerators (Varian 2100 and TrueBeam; Varian Medical Systems, Las Vegas, NV) for photon beams only. All of the beam data (both measured and calculated) used in this study were extracted from the beam modeling module of the TPS using a 2 mm fluence grid and a $50\;\times \;50\;{\text{cm}}^{2}$ phantom for computation. For the VS, the data are sampled using 100 data points across the geometric field size, and the phantom size is generated dynamically by adding an additional 11 voxels to each side of the field size. Both the voxel size and the sampling grid are dependent on the geometric field size. PDDs and profiles were then generated and extracted from the VS having the exact beam geometries as those in the beam modeling module of the TPS. Field sizes ranging from $1\;\times \;1\;{\text{cm}}^{2}$ to $40\;\times \;40\;{\text{cm}}^{2}$ were used in this analysis. The $1\;\times \;1\;{\text{cm}}^{2}$ and $2\;\times \;2\;{\text{cm}}^{2}$ small fields were generated/measured using the multileaf collimator (MLC) with an open jaw setting of $14\;\times \;14\;{\text{cm}}^{2}$. Field sizes $3\;\times \;3\;{\text{cm}}^{2}$ to $40\;\times \;40\;{\text{cm}}^{2}$ were measured/generated using only jaw settings with no MLC. All of the beam data were measured/calculated on a water phantom using an ion chamber (IC). Profiles were extracted at four depths, ranging from $D_{\text{max}}$ to 22 cm depth for 6x and 18x and five depths from $D_{\text{max}}$ to 30 cm for 15x. 15x had profiles at five depths because it was only recently that we commissioned this beam for clinical use, whereas the 6x and 18x beams had been commissioned previously. All PDD and profiles from the TPS and the VS were resampled at 1 mm intervals and were analyzed using in‐house software.

To analyze the PDDs, the curves were first renormalized to $D_{\text{max}}$. Using the measured data as the reference, the difference of TPS to measured data was computed for each data point. This was done from $D_{\text{max}}$ to a depth of 25 cm and an average PDD error was then computed for each field size. This process was repeated using the VS PDD for comparison to the measured data.

**Figure 1:**
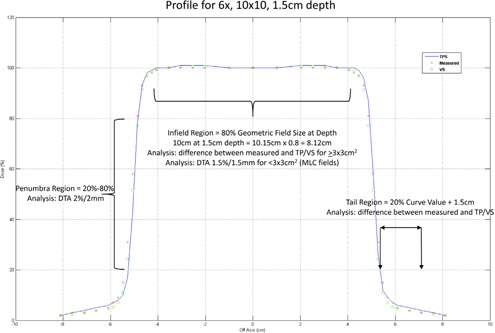
Illustration of profile analysis overlaid on a $10\;\times \;10\;{\text{cm}}^{2}$ field size measured at a depth of 1.5 cm for 6x photons.

To analyze the error in profiles, all profiles were first normalized to the central axis. Each profile was analyzed in three regions: in‐field, penumbra, and the tail region. The in‐field region is defined as the central 80% of the geometric field size projected at depth, the penumbra region is defined as the region bounded by 20% and 80% of the profile on each side, and the tail region is defined as the region between 20% on the curve to an additional 1.5 cm beyond this point on each side of the profile. Analysis of the in‐field data was done by taking the difference between measured and the TPS for each data point in the infield region for each profile. This difference for all data points of a specific field size (6x and 18x had four profiles and 15x had five profiles per field size) were grouped together and an average (and standard deviation) was computed and is referred to as the in‐field error. For the $1\;\times \;1\;{\text{cm}}^{2}$ and $2\;\times \;2\;{\text{cm}}^{2}$ fields (generated with the MLC), the in‐field data were analyzed using distance‐to‐agreement (DTA) metrics, with 1.5%/1.5 mm as the passing criteria. This was done by first determining the percentage of data points passing the DTA criteria per profile, and then computing the average DTA for each field size (averaged over multiple profiles acquired at different depths), and is referred to as the average in‐field DTA. This was done because for these two field sizes, there is no flat region and, basically, all the data points are within a gradient region. For the penumbra region, a DTA analysis was performed using 2%/2 mm agreement criteria, and the penumbral DTA was computed in the same fashion as the in‐field data. To analyze the tail region, the difference between the TPS and measured data was recorded, and this was averaged over all profile depths for a given field size. In addition to comparing the TPS to measured data, the same comparison was made using measured data as the reference to the VS. These analyses are illustrated in Fig. 1, which are the profiles from measured data, TPS, and VS for a 10 × 10 field size for a depth of 1.5 cm.

It is important to note that the VS can be fully operational using its own stock beam model provided in the software by inputting only one parameter, the PDD value at 10 cm depth in water per photon energy. If it is desired to further customize the stock beam model of the VS to better match the linear accelerator at an institution, this is accomplished by inputting several parameters for a limited number of field sizes/depths. These include PDD values at three depths, for three different field sizes, output factors for six different field sizes, and off‐axis ratios for a $40\;\times \;40\;{\text{cm}}^{2}$ at six different positions off‐axis for each photon energy. These parameters were entered into the VS to achieve a better match to our measured data and our TPS model. All of the new PDD and profile VS model information was extracted again for comparison. PDD results are presented with the stock VS beam model results, in addition to the adjusted VS beam model, while the profile results are only presented using the adjusted beam model. Output factors entered into the VS are also output by the VS (i.e., they fit the beam model to exactly what is input).

After customizing the VS beam model, 40 treatment plans for patients that were treated at our institution were retrospectively sent from the TPS to the VS to compute the dose. These patients were treated using step‐and‐shoot IMRT or VMAT. An ion chamber (CC04 ion chamber, Scanditronix, Wellhofer, Schuarzenbruck, Germany) and film (Kodak EDR2, Rochester, NY) were used to measure the dose delivered from the linear accelerator for comparison to the TPS and VS calculated dose. Dose was delivered to a rectangular solid water phantom (built in‐house) that has inserts for ion chambers and a cassette for film. Point dose was extracted from each of the plans, and this was compared to point dose calculated in the TPS and to dose measured using an IC. The point dose was determined in the TPS and VS by placing a region of interest (ROI) with the same geometry as our ion chamber and recording the average dose to this ROI. The ratio of TPS dose/IC dose, VS Dose/IC dose, and TPS dose/VS dose was computed for each patient and tabulated. An average of each of these ratios was tabulated for all cases, and then separated to compare IMRT and VMAT specific cases. A dose plane was extracted from both the TPS and the VS in the same geometry as our phantom, and a gamma analysis was performed to determine the number of pixels passing gamma at the 3%/3 mm criteria (DoseLab, Mobius Medical Systems, Houston, TX). The following planar dose comparisons were made, VS to TPS, VS to film, and TPS to film. The average percentage pixels passing gamma was determined for each comparison for all 40 patients analyzed. An overall average was then computed for all the planar dose analysis.

## III. RESULTS & DISCUSSION

PDDs and profiles were compared from the VS and the TPS and to measured data. The average PDD errors relative to measured data for the TPS, the stock VS beam model, and the adjusted VS beam model for 6x, 15x, and 18x are shown in 2, 3, 4, respectively. For 6x, the TPS and VS on average is less than 0.21% and 0.87%, respectively, from our measured data for field sizes larger than $2\;\times \;2\;{\text{cm}}^{2}$. For the 1 × 1 and $2\;\times \;2\;{\text{cm}}^{2}$ field sizes, the TPS on average was less than 0.35% and 2.28% from measured data and from the VS, respectively. For 15x, the TPS and VS on average is less than 0.29% and 1.38%, respectively, from our measured data for field sizes larger than $2\;\times \;2\;{\text{cm}}^{2}$. For the 1 × 1 and $2\;\times \;2\;{\text{cm}}^{2}$ field sizes, the TPS on average was less than 0.42% and 1.02% from measured data and from the VS, respectively. For 18x, the TPS and VS on average is less than 0.12% and 1.07%, respectively, from our measured data for field sizes larger than $2\;\times \;2\;{\text{cm}}^{2}$. For the 1 × 1 and $2\;\times \;2\;{\text{cm}}^{2}$ field sizes, the TPS on average was less than 0.52% and 2.23% from measured data and from the VS, respectively.

**Figure 2:**
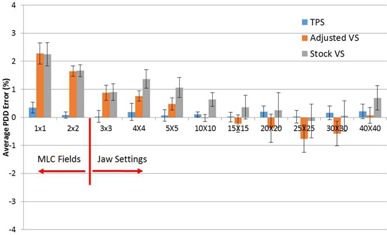
The average PDD error relative to measured data for 6x photons.

**Figure 3:**
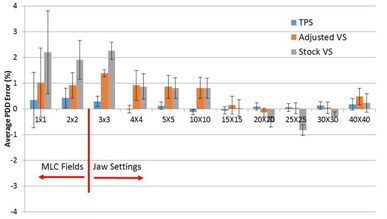
The average PDD error relative to measured data for 15x photons.

**Figure 4:**
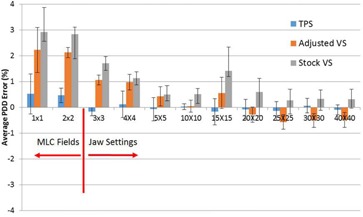
The average PDD error relative to measured data for 18x photons.

Shown in 1, 2, 3 are results from comparison of profiles to our measured data for 6x, 15x, and 18x profiles for the field sizes generated using jaw settings. For 6x, the worst case average in‐field error was 0.98% and 0.58% for the TPS and VS, respectively, to measured data, both occurring at the $3\;\times \;3\;{\text{cm}}^{2}$ jaw settings. All other field sizes had errors less than 0.25% and 0.35% for the TPS and the VS, respectively. For 15x, the average in‐field errors ranged from 0%–0.37% for the TPS and −0.01%–0.37% for the VS. For 18x, the average in‐field errors ranged from 0.01%–0.7% for the TPS and 0%–0.77% for the VS. For the penumbra region, 100% of points passed the DTA criteria for field sizes less than $10\;\times \;10\;{\text{cm}}^{2}$ for both the TPS and the VS for 6x. Larger field sizes resulted in fewer points passing the 2%/2 mm DTA criteria. High‐energy photons yielded more penumbral points passing the DTA criteria for both the TPS and the VS. The average out‐of‐field profile error for 6x for the TPS ranged from −0.68%—1.5%, while the VS ranged from 0.69%—4.49%. For 15x, the average out‐of‐field error ranged from 0.29%—1% for the TPS and −0.02%—3.02% for the VS.

**Table 1:** 6x profile average error summary relative to measured data for jaw settings

	*In‐field*		*% Passing Penumbral DTA*		*Out of Field*	
*Field Size*	*TPS % Diff*.	*VS % Diff*.	*TPS*	*VS*	*TPS % Diff*.	*VS % Diff*.
3×3	0.98±1.42	0.58±1.30	100.00±0.0	100.00±0.0	‐1.04±0.8	0.69±1.2
4×4	‐0.04±0.35	‐0.09±0.37	100.00±0.0	100.00±0.0	‐1.50±1.2	‐1.17±0.7
5×5	0.05±0.46	‐0.35±0.55	100.00±0.0	100.00±0.0	‐1.44±1.1	‐1.39±0.8
10×10	‐0.07±0.29	‐0.05±0.46	92.00±0.2	90.00±0.2	‐1.23±1.0	‐2.06±0.7
15×15	‐0.25±0.32	‐0.00±0.43	85.79±0.2	66.82±0.2	‐1.16±1.1	‐4.04±0.9
20×20	‐0.17±0.35	‐0.19±0.51	78.77±0.3	80.78±0.3	‐1.09±1.2	‐2.48±0.6
25×25	‐0.06±0.33	‐0.21±0.49	71.60±0.3	52.86±0.2	‐1.02±1.3	‐4.49±0.8
30×30	‐0.11±‐0.34	‐0.25±0.44	62.00±0.3	66.48±0.2	‐0.68±1.3	‐2.49±0.7
40×40	‐0.00±0.45	‐0.01±0.45	24.86±0.2	^a^	^a^	^a^

a Analysis was not completed because of lack of data.

**Table 2:** 15x profile average error summary relative to measured data for jaw settings

	*In‐field*		*% Passing Penumbral DTA*		*Out of Field*	
*Field Size*	*TPS % Diff*.	*VS % Diff*.	*TPS*	*VS*	*TPS % Diff*.	*VS % Diff*.
3×3	0.16±0.73	0.61±0.88	100.00±0.0	100.00±0.0	0.29±0.5	1.48±0.6
4×4	0.05±0.75	0.21±0.79	100.00±0.0	100.00±0.0	‐0.89±1.1	0.50±1.0
5×5	0.00±0.45	‐0.34±0.55	100.00±0.0	100.00±0.0	‐0.32±0.8	1.21±0.8
10×10	0.37±0.53	‐0.14±0.36	100.00±0.0	100.00±0.0	‐0.36±0.8	1.03±1.1
15×15	0.26±0.46	‐0.04±0.46	100.00±0.0	94.16±0.1	‐1.00±1.1	‐2.53±1.4
20×20	0.21±0.43	0.08±0.45	98.44±0.0	100.00±0.0	‐0.66±1.1	0.10±1.3
25×25	0.01±0.35	‐0.13±0.39	96.88±0.1	73.75±0.1	‐0.78±1.4	‐3.02±1.6
30×30	0.21±0.36	0.20±0.30	83.61±0.1	93.75±0.1	‐0.96±1.4	‐0.02±1.2
40×40	‐0.12±0.50	‐0.01±0.60	14.29±0.3	^a^	^a^	^a^

a Analysis was not completed because of lack of data.

Shown in Table 4 are results from the profile analysis for 6x, 15x, and 18x for $1\;\times \;1\;{\text{cm}}^{2}$ and $2\;\times \;2\;{\text{cm}}^{2}$ field sizes generated using the MLC. For the in‐field analysis, a 1.5%/1.5 mm DTA analysis was performed. This was done because 100% of in‐field data points for both the TPS and the VS passed at the 2%/2 mm criteria, so the criteria was tightened to observe any differences. Please note that the penumbra DTA analysis was kept at 2%/2 mm. We see for the $1\;\times \;1\;{\text{cm}}^{2}$ field size, 100% of the in‐field points pass meet the DTA criteria for all energies for the TPS, while for the VS, this is true for high‐energy photons. For 6x, $1\;\times \;1\;{\text{cm}}^{2}$ field size for the VS, 78.9% of in‐field data points passed the 1.5%/1.5 mm criteria. For the $2\;\times \;2\;{\text{cm}}^{2}$ field size, 96.1% of pixels passed criteria for 6x and 100% passed for high‐energy photons computed by the TPS. The VS had a slightly lower number of points passing criteria for the $2\;\times \;2\;{\text{cm}}^{2}$ field size. Excellent agreement was observed in the penumbra regions for both the TPS and the VS, with all data points passing DTA criteria, with the following exceptions: 96% of penumbra passed for the TPS for the 15x, $2\;\times \;2\;{\text{cm}}^{2}$ and similar results were observed for 6x, $2\;\times \;2\;{\text{cm}}^{2}$ for the VS. Out‐of‐field errors ranged from −0.67%—2.01% for the TPS and 0.57%—1.78% for the VS.

Ion chamber results were compared to both the TPS and the VS for 40 patients for IMRT and VMAT cases. Shown in Table 5 is the distribution of anatomical sites for patients used in this study. The ion chamber results are summarized in Table 6, which shows the average ratio of TPS or VS to ion chamber and TPS to VS for all 40 patients. Also shown in this table are the cases separated by IMRT or VMAT plans. In this analysis, 12 cases were VMAT plans and 28 cases were step‐and‐shoot IMRT plans. From this data, we see a slightly better agreement to our ion chamber measurements from the VS relative to the TPS for all cases with the ratio of TPS/IC and VS/IC dose on average of 100.5% ± 1.9% and 100.4% ± 1.3%, respectively. For the VMAT cases, an average ratio of TPS/IC and VS/IC dose was 102.2% ± 1.2% and 101.5% ± 1.0%, respectively, and for IMRT cases was 99.8% ± 1.7% and 99.8% ± 1.8%, respectively. The planar dose analysis (Table 7) was performed and compared to both film and the TPS to the VS. The average percent pixels passing gamma for 40 patients of the VS to film was 97% ± 5.6%, slightly higher than the results from our TPS to film, which was 96.6% ± 4.2%, while comparing the VS to the TPS, on average 98.1% ± 5.3% pixels passed the gamma analysis. This exceeds our clinical passing criteria for planar dose analysis, which is 90%.

**Table 3:** 18x profile average error summary relative to measured data for jaw settings

	*In‐field*		*% Passing Penumbral DTA*		*Out of Field*	
*Field Size*	*TPS % Diff*.	*VS % Diff*.	*TPS*	*VS*	*TPS % Diff*.	*VS % Diff*.
3×3	0.48±0.66	0.77±1.12	100.00±0.0	100.00±0.0	0.21±0.3	1.30±0.8
4×4	0.70±1.06	0.41±0.92	100.00±0.0	100.00±0.0	‐0.61±1.7	‐0.62±1.5
5×5	0.25±0.54	‐0.08±0.66	100.00±0.0	100.00±0.0	0.05±1.1	‐0.13±0.9
10×10	0.29±0.38	0.26±0.39	100.00±0.0	100.00±0.0	‐0.09±1.0	‐0.87±0.9
15×15	0.43±0.44	0.16±0.37	100.00±0.0	100.00±0.0	0.09±1.0	‐2.07±0.8
20×20	0.28±0.42	0.00±0.38	95.00±0.1	100.00±0.0	0.12±0.9	‐1.14±0.6
25×25	0.01±0.40	‐0.27±0.48	82.78±0.2	73.93±0.2	‐0.20±1.1	‐3.25±1.1
30×30	0.09±0.47	‐0.08±0.39	86.70±0.2	83.56±0.2	‐0.07±1.3	‐2.46±0.7
40×40	‐0.06±0.43	‐0.42±0.85	54.52±0.1	^a^	^a^	^a^

a Analysis was not completed because of lack of data.

**Table 4:** Profile average error summary relative to measured data for MLC field sizes

		*% Passing In‐field DTA*		*% Passing Penumbral DTA*		*Out of Field*	
*Energy*	*Field Size*	*TPS* ^a^	*VS* ^a^	*TPS* ^b^	*VS* ^b^	*TPS % Diff*	*VS % Diff*
6	1×1	100.00±0.0	78.99±0.1	100.00±0.0	100.00±0.0	‐1.23±1.0	0.70±2.5
15	1×1	100.00±0.0	100.00±0.0	100.00±0.0	100.00±0.0	‐2.01±1.8	‐1.78±3.6
18	1×1	100.00±0.0	100.00±0.0	100.00±0.0	100.00±0.0	‐1.01±0.4	0.57±2.0
6	2×2	96.09±0.1	67.84±0.1	100.00±0.0	96.00±0.1	‐0.67±0.8	1.60±2.5
15	2×2	100.00±0.0	100.00±0.0	96.00±0.1	100.00±0.0	‐1.63±1.6	‐0.87±3.3
18	2×2	100.00±0.0	86.02±0.2	100.00±0.0	100.00±0.0	‐0.83±0.4	1.65±2.1

a 1.5%/1.5 mm criteria.

b 2%/2 mm criteria

**Table 5:** Distribution of cases for dose comparison analysis

*Site*	*Number*
Thoracic	15
Gastrointestinal	9
Genitourinary	4
Head and Neck	12

**Table 6:** Point dose comparison for IMRT and VMAT cases (%)

	*TPS/IC*	*VS/IC*	*TPS/VS*
Total (n=40)	100.5±1.9	100.4±1.3	100.1±1.0
VMAT (n=12)	102.2±1.2	101.5±1.0	100.7±0.8
IMRT (n=28)	99.8±1.7	99.8±1.0	99.9±0.9

**Table 7:** Average % pixels passing gamma analysis (3%/3 mm) for IMRT and VMAT cases

TPS to Film	96.6±4.2
VS to Film	97.0±5.6
VS to TPS	98.1±5.3

## IV. CONCLUSIONS

In this study, we have presented the preliminary steps needed to commission a system that will be used as a secondary verification tool of a clinical TPS using a fully automated computer system. Our analysis was limited to photon beams produced from Varian accelerators. No analysis was performed on flattening filter‐free photon beams, electron beams, wedged beams, and beams for Tomotherapy machines. In this commissioning project, we have verified that the VS agrees with our measured data, and that we can rely on the system to accurately compute dose to a homogeneous medium for simple and complex geometry cases. In addition, excellent agreement is observed for point‐ and planar‐dose comparison for both step‐and‐shoot IMRT and VMAT treatment beams. We also have shown that adjustments can be made to the stock VS beam model, and the automodeling algorithm is able to adjust the beam model to better fit the characteristics of the treatment machine. Thus, the VS can accurately be used as a secondary check of the TPS.

The interface for this tool is very user friendly, and everything is done automatically once the DICOM RT export happens from the TPS. Before clinical release, further dose validation in heterogeneous mediums is necessary. Other areas of this system that need to be examined are the validation of the DVH comparison and the 3D gamma comparison. Plan checking capabilities need to be validated by introducing known errors and determining if the system is capable of detecting such errors. Our current 2D second check program only computes dose at a single point per beam, and it is only capable of doing so for simple geometries and IMRT beams, not VMAT beams. This system compares dose in the entire treated volume and performs a 3D gamma analysis of the difference between the TPS and VS dose calculation for photon beams only. While this gives us much more information than we presently have from a secondary verification standpoint, interpretation of this data initially can be overwhelming, and guidelines and tolerances must be established before clinical release.

## Supporting information

Supplementary Material

Supplementary Material

Supplementary Material

Supplementary Material

Supplementary Material
