# Supplementary figures and images for: Commissioning results of an automated treatment planning verification system
Source: J Appl Clin Med Phys. 2014 Sep 8;15(5):57–65. doi: 10.1120/jacmp.v15i5.4838 (PMC5711088; doi:10.1120/jacmp.v15i5.4838)

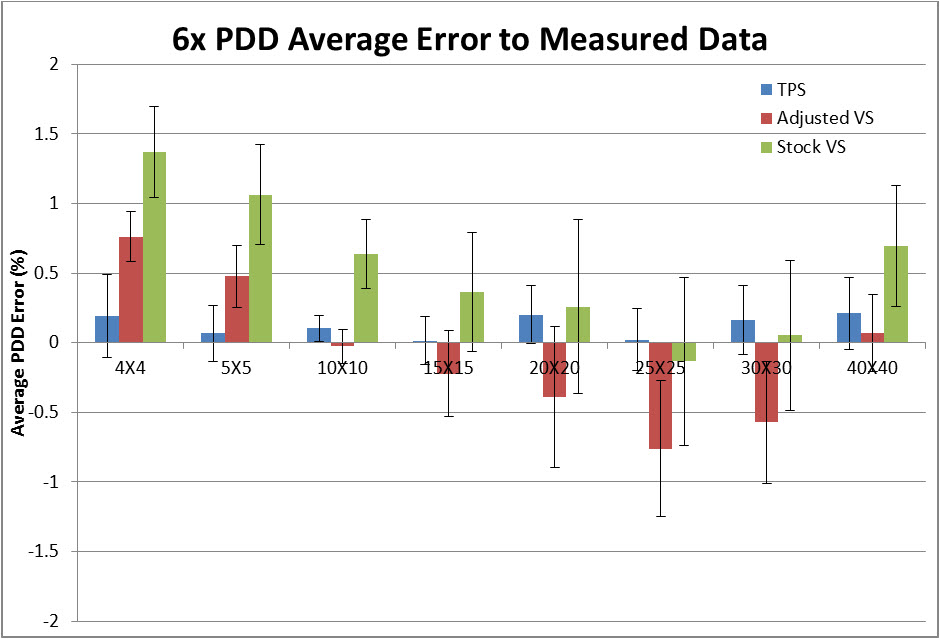

Supplement: Supplementary file 1 — Supplementary Material [file ACM2-15-057-s001.jpg]

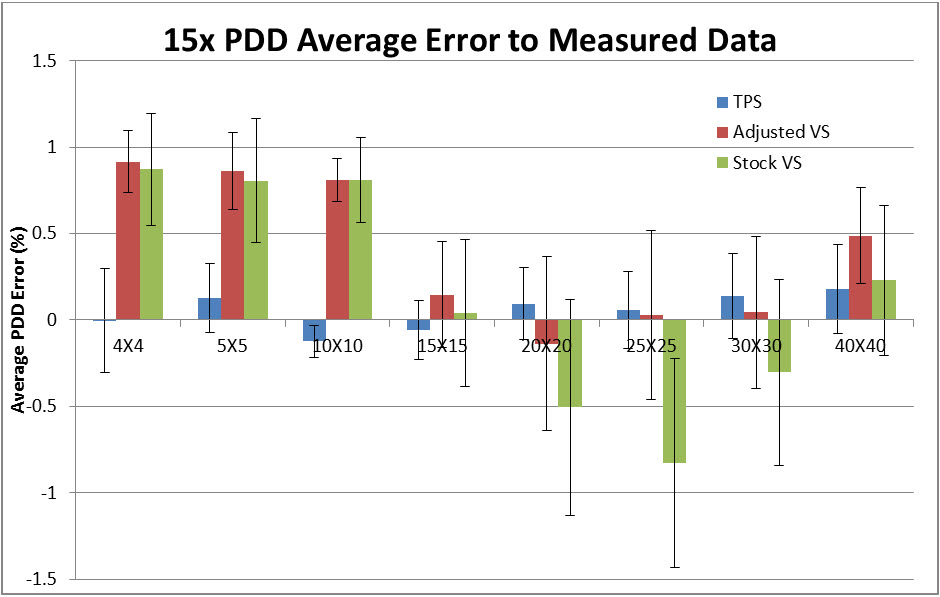

Supplement: Supplementary file 2 — Supplementary Material [file ACM2-15-057-s002.jpg]

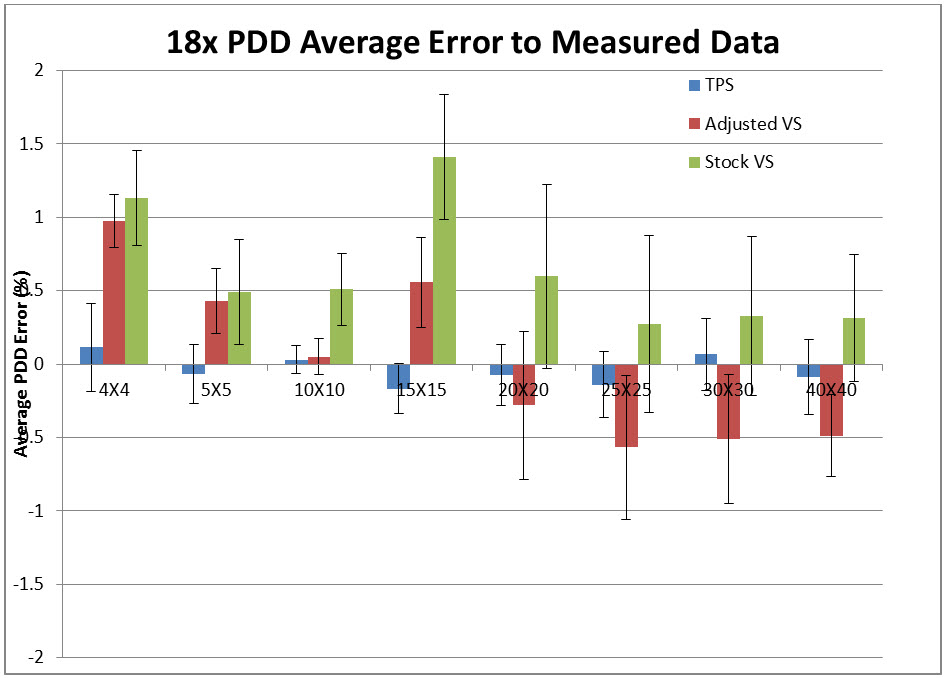

Supplement: Supplementary file 3 — Supplementary Material [file ACM2-15-057-s003.jpg]

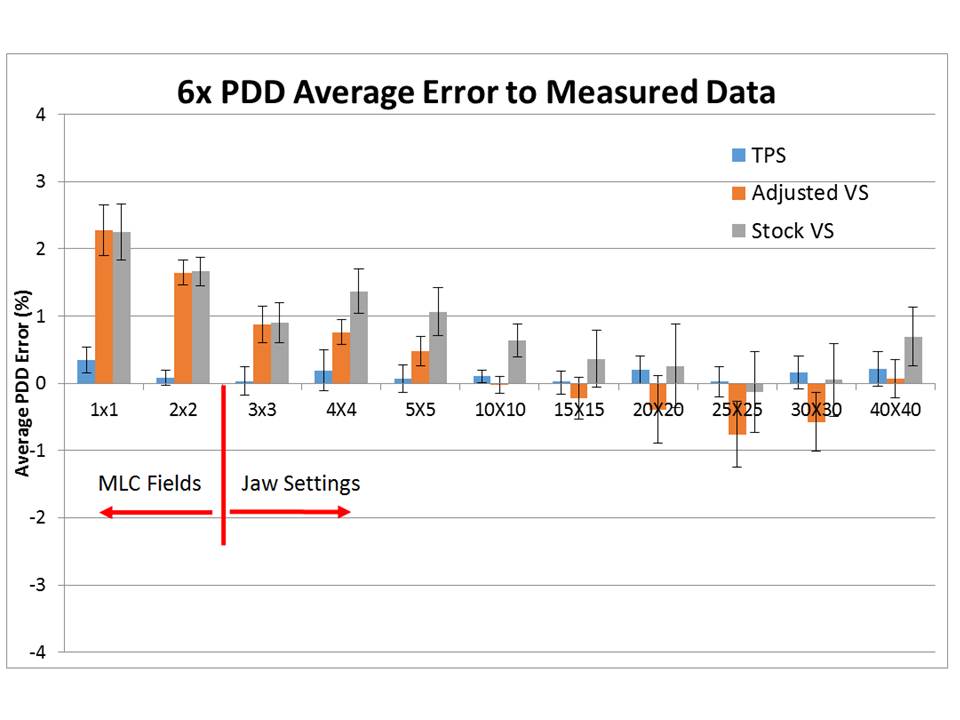

Supplement: Supplementary file 4 — Supplementary Material [file ACM2-15-057-s004.JPG]

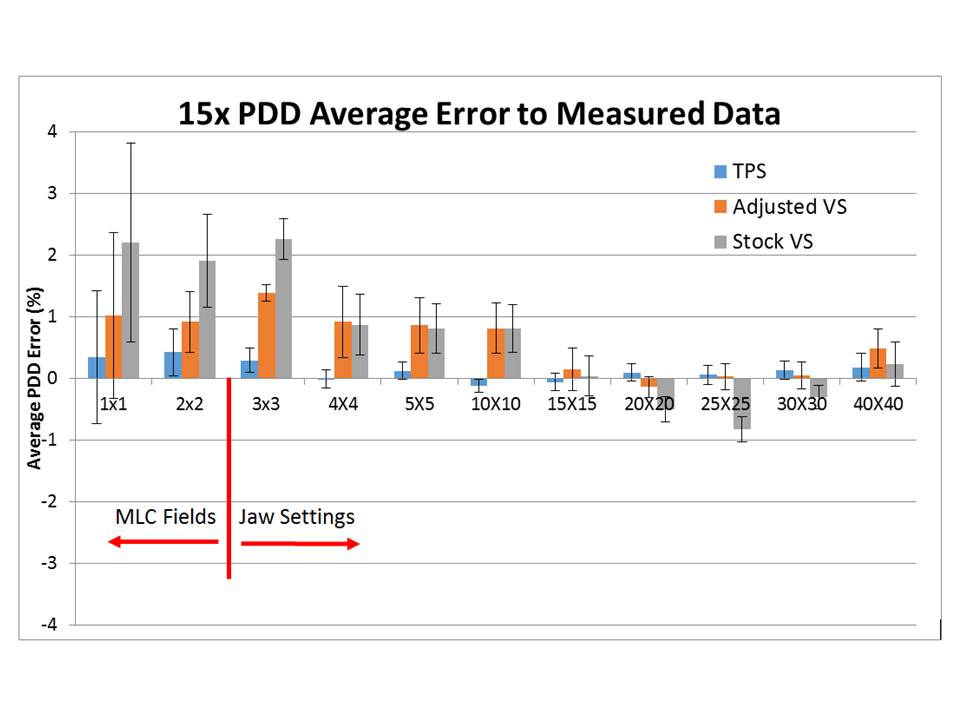

Supplement: Supplementary file 5 — Supplementary Material [file ACM2-15-057-s005.JPG]
